# Participatory Systems Mapping for Municipal Prioritization and Planning

**DOI:** 10.1007/s11524-022-00654-2

**Published:** 2022-07-07

**Authors:** Amanda Pomeroy–Stevens, Bailey Goldman, Karen Grattan

**Affiliations:** 1 Building Healthy Cities Project, JSI Research & Training Institute, Inc, Arlington, VA USA; 2 Building Healthy Cities Project, Engaging Inquiry LLC, Durham, NC USA

**Keywords:** Systems thinking, Systems mapping, Urban systems, Participatory methods, Citizen participation

## Abstract

Rapidly growing cities face new and compounding health challenges, leading governments and donors to seek innovative ways to support healthier, more resilient urban growth. One such approach is the systems mapping process developed by Engaging Inquiry (EI) for the USAID-funded Building Healthy Cities project (BHC) in four cities in Asia. This paper provides details on the theory and methods of the process. While systems mapping is not new, the approach detailed in this paper has been uniquely adapted to the purpose of municipal planning. Strategic stakeholder engagement, including participatory workshops with a diverse group of stakeholders, is at the core of this approach and led to deeper insights, greater buy-in, and shared understanding of the city’s unique opportunities and challenges. This innovative mapping process is a powerful tool for defining municipal priorities within growing cities across the globe, where the situation is rapidly evolving. It can be used to provide evidence-based information on where to invest to gain the biggest impact on specific goals. This paper is part of a collection in this issue providing a detailed accounting of BHC’s systems mapping approach across four project cities.

## Introduction

Rapidly growing cities face new and compounding health challenges such as pollution, inequitable access to services, and insufficient physical infrastructure. Climate change exacerbates these issues, particularly for the urban poor [1]. There is ample evidence that, compared to rural areas, people in urban settings face an increased inequity in access to services and higher risk for noncommunicable diseases, traumatic injuries, and certain infectious diseases [2–4]. There are knowledge gaps on cost-effective ways to control disease and protect the most vulnerable city dwellers [5].

Governments and donors are actively seeking ways to support healthier, more resilient urban growth. They also acknowledge that cities present different challenges than rural or mixed growth settings. This complexity challenges traditional policy solutions that apply one policy to solve one problem in one sector. Urban settings require cross-sectoral, nonlinear approaches that engage a diverse set of stakeholders. Defining key stakeholders is always tricky, and previous urban planning approaches uncovered the pitfalls of not including the right mix [6–8]. When done in an equitable manner, early stakeholder engagement increases transparency, creates more relevant solutions, allows for earlier identification of available resources, and generates greater commitment to urban health-strengthening efforts.

The United States Agency for International Development (USAID)–funded Building Healthy Cities project (BHC) is testing what works well for healthier urban planning in four rapidly growing Smart Cities in Asia. JSI Research & Training Institute, Inc. (JSI) leads BHC with partners International Organization for Migration, Thrive Networks, and Urban Institute. JSI hired Engaging Inquiry (EI), a global leader in participatory, systemic action inquiry, to support BHC’s efforts to use systems thinking as an organizing framework for healthier cities. This paper provides details on the theory and methods of the systems mapping process that EI developed for BHC to use in its partner cities.

### Origins of Systems Mapping

Systems mapping combines elements from three fields of practice:

- Quantitative mapping of structural factors and processes. Quantitative mapping pulls from systems dynamics, an approach to understanding the nonlinear behavior in systems which originated in the fields of industrial organization and business [9, 10]. The resulting diagrams provide a conceptual model of a closed system that is populated with quantitative data to define relationships. This area of work helped to define circular, or loop (rather than linear), diagramming and focused on creating a shared model for making production or management decisions [11]. While this approach represented a major step forward from strictly linear models, using only empirically proven relationships severely limits the representativeness of the maps, particularly for topics without ample quantitative evidence.
- Systems thinking theory. There is a wealth of literature on applying systems thinking to health [12–16]. This work typically relates to health systems strengthening and can be useful for conceptualizing what is included in a system. However, systems thinking has struggled to gain traction in implementation and real-world use [17–20]. Relevant toolkits typically focus on changing “thinking” (knowledge and attitudes) and stop short of creating practical and reproducible processes, or defining measureable outputs to guide implementation, engagement, and learning [21].
- Community-based participatory methodologies. Truly inclusive, participatory approaches for health are crucial in order to develop relevant, inclusive solutions [22–24]. However, after a series of potentially exploitative studies were conducted under the guise of participatory research [25–28], sentiment turned away from participatory approaches. Even when these approaches are applied well and results are truly mutually beneficial, a common constraint is that these processes do not connect back to the local power brokers in a way that can turn the results into funded, implemented actions [22].

EI’s systems approach incorporates the strengths of each of these bodies of work, while addressing the known shortcomings. It provides (a) a visual tool for building shared understanding of complex systems; (b) a mutually beneficial listening and decision-making structure that builds from the community up to the government and donor level; (c) a method for identifying where change is most likely to happen in the system; and (d) a more structured and transparent process for developing next steps and identifying resources. The resulting recommended actions can be tested quantitatively if desired, as with any theoretical model. EI’s approach was originally developed to support philanthropic sector programs in making high-impact strategic investments [29]. Later, EI adapted its practice to support coalition building and strategy development funded by the US government and local health departments [30–32].

EI and BHC are not the only ones adapting systems approaches to urban health. Most applications focus on defining theories or concepts for addressing urban health systems [33] and/or developing indicators to measure systems (or the related health-in-all-policies) approaches to urban health [34–40]. The studies most similar to our work were undertaken by the Urban Health in Latin America (SALURBAL) project across eight countries. SALURBAL completed multiple systems thinking activities that looked at urban food and transport systems, using systems mapping exercises including the cross-impact balance method (which draws out expert feedback on a set of factors, using a bivariate ranking system) and community-based participatory workshops which developed causal loop diagrams [41–44]. System dynamics models have also been used to better understand the drivers of obesity, though not always specifically for urban settings [45, 46]. The UK Government’s Foresight Programme developed a comprehensive atlas of the systems relating to childhood obesity in 2008, but some critiques suggested the results were too complex to be put to practical use [47, 48].

The approach developed for BHC by EI is unique in our use of the systems mapping process as an implementation framework for an urban health systems strengthening program. This methodology is particularly well suited to this task because of its focus on transparency, inclusiveness, mutual benefit, and actionable outputs. This approach complemented BHC’s other systems thinking work, which included using visual tools to define progress over time; developing multi-sector working groups to bring together a wide range of stakeholders; integrating data across disparate sectors to support informed decision-making; and participatory research activities [49–51].

Other articles in this issue describe how BHC put this methodology to use in three of our partner cities [52–54]. Here, we describe the specific elements of the systems mapping process.

## Methods

**Fig. 1:**
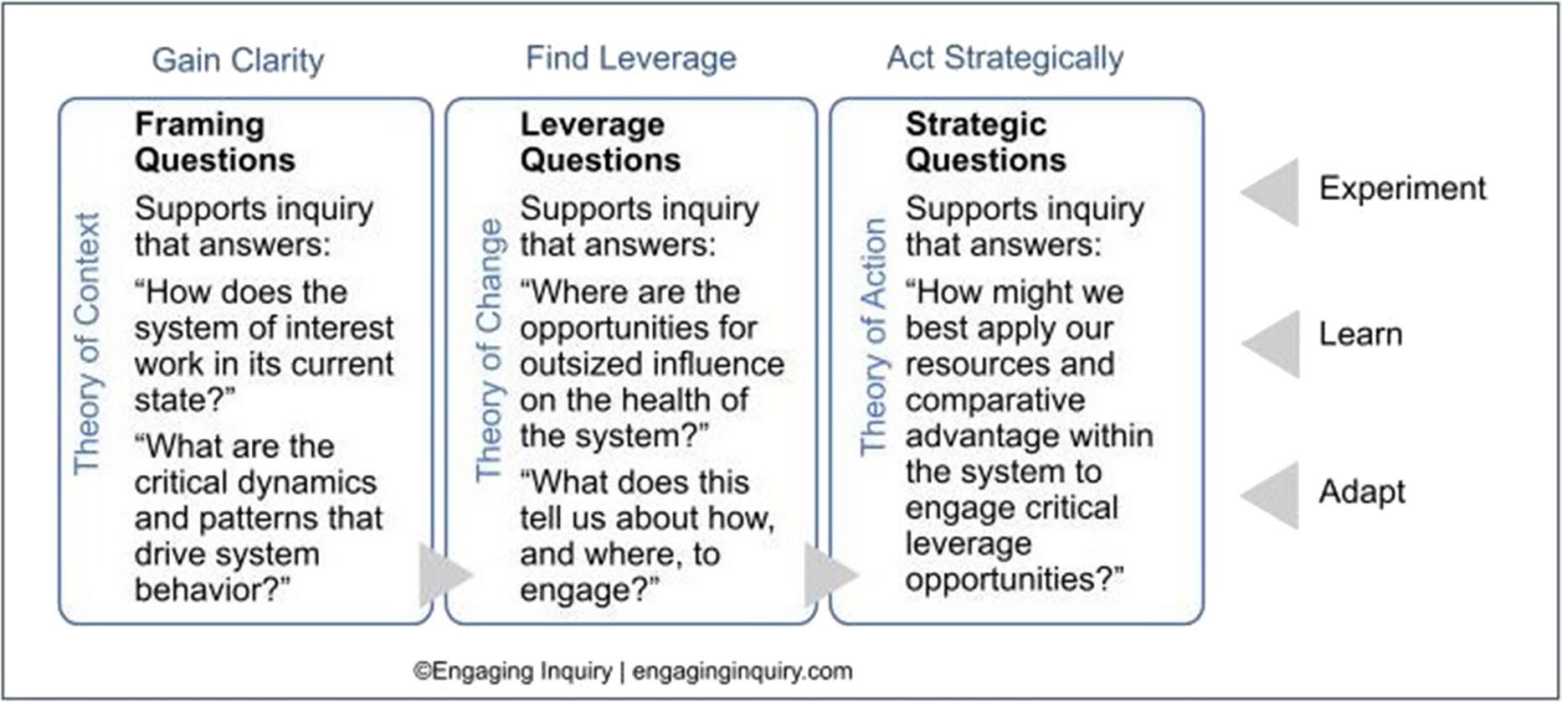
Three phases of systems mapping

EI and BHC’s systems mapping approach is characterized by three steps (context, change, and action) described in Fig. 1 and evolved from grounded theory methods.

This version of systems mapping was based on primary qualitative data collected via participatory methods (learning sessions and stakeholder engagement workshops) as well as focus group discussions, key informant interviews, and direct observation. The data were used to build causal loop diagrams (“maps”), define inter-connections between issues, and develop narrative descriptions for each loop, opportunity, and proposed action. Each map, when looked at in total, can then provide a detailed description of how things currently work and how things might change to improve the overall health of the city and its citizens. A valuable facet of this approach is its ability to absorb outside data—BHC collected quantitative and qualitative data in each city outside of the systems mapping, and for each set of results, BHC revisited the maps with the new evidence and was able to adjust the descriptions based on these findings. This allowed for triangulation and verification of recommendations and forced BHC to check the system maps for any unexpected interactions or consequences. In this way, the maps were established as living, learning tools.

Typical application of the systems mapping runs between 9 and 12 months in duration. This process is designed to be flexible and can be adapted to different contexts. Elements such as funding or election cycles, sharing opportunities, or project due dates can all be incorporated to develop the most effective timeline.

### Participant Sampling

EI generally suggests a purposive sampling approach targeting participants necessary for achieving (1) a diverse range of informed perspectives on how the city system currently operates, and (2) uptake and support of the final recommendations. Purposive sampling can create bias if it does not recognize some key groups, so EI suggests including the sectors, demographics, and positions needed to accurately describe the structures, behaviors, and mindsets driving current system outcomes. BHC used existing baseline data and relationships with each city to ensure we engaged the right sectors, while also aiming for representation from each of the following: government, civil society, academia, donors, and the private sector. While the number of participants depended on the city, EI suggests an ideal participation rate of 25–40 for each workshop to maximize involvement in the focus group discussions. Since BHC’s maps were living documents, adding new stakeholders throughout the process was allowed if they brought new, relevant perspectives.

BHC also organized a parallel set of citizen engagement workshops called town halls, with groups identified during baseline assessments as vulnerable populations within each city. Their participation was requested separately from the main workshops to give citizens a forum where they did not feel their privacy or freedom to speak might be threatened by those in positions of power.

### Prepositioning: Defining the Domain of Each System

Before the workshops began, EI led the BHC team in each city through a series of discussions to:

1. Define the desired outcome, or “Guiding Star” and “Vision Statement.” Establishing a shared definition of success is the essential first step toward creating a stronger system. The best definitions strike a good balance between inspiring and concrete. Defining a Guiding Star enabled BHC to create a shared definition of what it means to have a healthy system. The team then translated this to a Vision Statement, which describes the desired goal of the system. In the city context, there is often a slogan that can be adapted for this purpose—in BHC’s case, this was usually the Smart City initiative’s slogan or goal statement. Adapting this existing material demonstrated BHC’s commitment to supporting existing city goals (as opposed to criticizing or competing with them) and helped to garner trust from city officials. BHC used the final Vision Statement to keep participants on track throughout the 3-step process.
2. Frame the system of interest. For the purposes of this work, a system is defined as a diverse set of parts that interact with each other and their environment in ways that are dynamic and often hard to predict, but can be mapped, understood, and influenced. The system boundaries are not pre-defined (as in, the healthcare system, education system, elections system, etc.). EI coached BHC city teams to frame their system boundaries by asking: *what is it that we, given our position and potential, most need to know about the current system so that we can understand what is needed to move it closer to the Guiding Star?*

### Step #1: Defining Context—Data Collection and Analysis Techniques

BHC convened a participatory workshop in each city to collect the qualitative data used to create the building blocks for the first system map: a Theory of Context. Where BHC had done extensive baseline qualitative assessments prior to the Context Workshop, EI coached the BHC team to build from that data for this first step (described in more detail by our Indore and Makassar papers in this issue) [52, 53]. In Da Nang, Vietnam, where BHC had not collected baseline data, the first step was to ask workshop participants to reflect on their own experiences to list key forces that were either “inhibiting” the system from producing health, or “enabling” it to produce health [54]. In both formats for this workshop, participants where then asked to prioritize these forces.

**Fig. 2:**
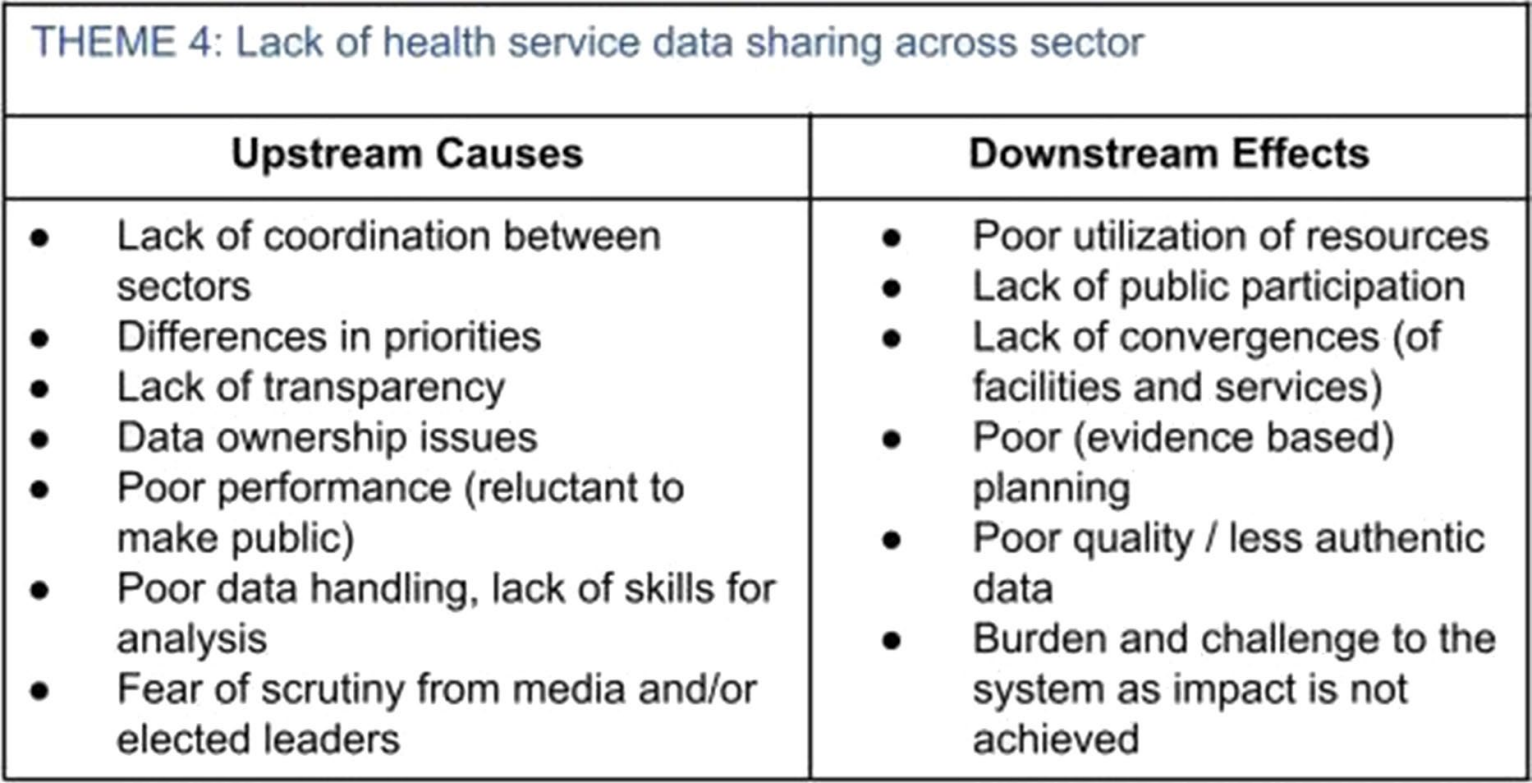
T-chart for defining upstream and downstream effects

Following the identification step, the key system forces were organized around themes. Participants were broken into small groups of three to conduct a cause and effect analysis on each theme. Each group was given a simple T-chart on poster paper, with their selected theme written at the top. Figure 2 shows one completed example from the BHC city of Indore.

Participants brainstormed and recorded first the causes, or “upstream” factors, that lead to the existence of that force in the system, and then the effects, or “downstream” factors, that this force creates. This was not about consensus, but instead uncovering the diverse perspectives and experiences across the system. Participants were encouraged to check the depth of their analysis through a Structural, Attitudinal, and Transactional (SAT) Framework, provided as a reference during the workshop.

**Fig. 3:**
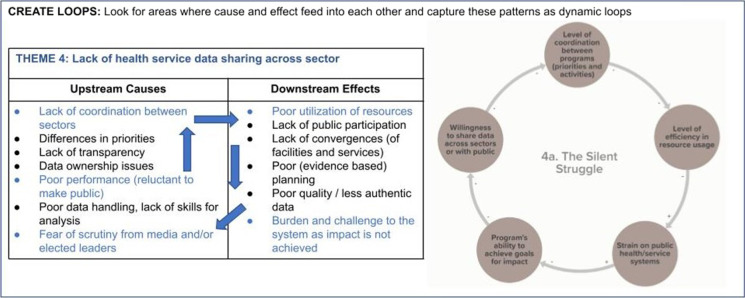
Example of participatory causal loop building

Next, feedback loops were built from these worksheets. These loops provide a powerful tool to understand how different issues may have non-linear relationships with each other. Participants were asked to identify these feedback patterns and build them into causal loops, facilitated by EI and BHC staff (Fig. 3). Having participants build the loops made the final map more reflective of the real system and more readily understood and adopted by key stakeholders.

After the workshop, EI and BHC city teams finalized the loops to develop the context map. EI and BHC used Kumu, an online systems mapping software, to house the data and loops [55]. This platform (with both free and paid options) creates public access to the maps and allowed the team to be transparent in data tracking, storage, and analysis.

To finalize analysis, the team first categorized the causal feedback loops developed in the workshop as either reinforcing or balancing. Reinforcing loops can either be vicious (negative) or virtuous (positive), but they are always self-perpetuating. Balancing loops either stop or limit the initial element, by stabilizing (where something begins as a negative but is mitigated by a positive force) or stagnating (where something begins as a positive but is disrupted by a negative force). Loops are made with factors (bubbles) connected by arrows to indicate the causal relationship. Beside each factor’s connecting arrow is a value sign indicating whether the level of that factor in the system is high/increasing ( +) or low/decreasing (-) (these signs do not denote good or bad).

Next, the team connected the loops to create the full system map. The team defined which loops, key elements, or concepts were repeated most frequently to create a central loop called the “Deep Structure.” This loop holds together the rest of the map. Around the Deep Structure, additional loops are carefully interwoven to reflect the behaviors, interactions, and conflicts identified in the workshop.

The first comprehensive draft system map, the “Provisional Map,” was shared during the community town halls. In small group discussions, participants were asked what most resonated with them and their own experiences, what was surprising or confusing, and what needed to be further explained. Their feedback was then incorporated into the map and each loop narrative. In this way, the context map was always in some state of collection or analysis. Once all essential perspectives were included, it became a “Working Map” and the team moved into the next phase of work, Leveraging.

### Step #2: Finding Leverage—Data Collection and Analysis Techniques

The context map served as a tool to identify the best opportunities for impact. Stakeholders were once again convened in a workshop to review the working context map and complete small group data collection and initial analysis. Participants were asked: *what is this system functioning perfectly to produce?* This question defines where the system is as of the workshop, and opens inquiry into what must change to move it closer to the desired outcome.

**Fig. 4:**
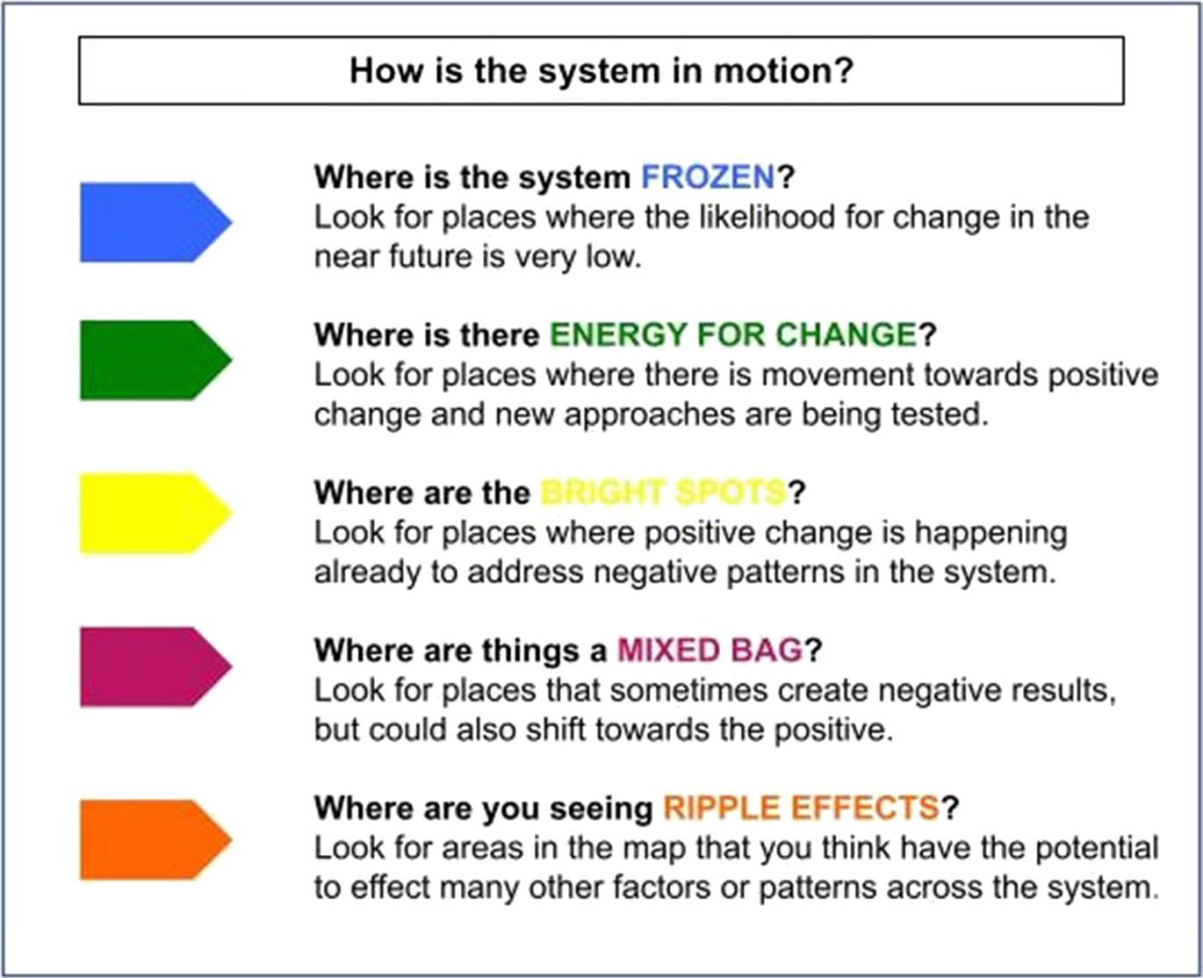
Leverage exercise

During small group work, participants marked the factors in the working context map with colored “flags” to identify types and location of energy in the system (described in Fig. 4). Because factors are framed at the system level, a factor can reflect different types of energy at different levels of analysis.

Next, looking across the flags on the map, groups identified areas where bright spots and energy were clustered and chose which ones they saw as having the greatest potential to shift important behaviors or relationships in the system. Together, they worked through the following analysis process, which is always grounded in what is observed in the map:

1. What area do we identify as holding high-leverage potential for change in the system?
2. So what can we learn, and apply, from what is/has already happened here?
3. What if we could create this shift? What are the direct and proximal impacts we would expect to see (if–then logic within the map)?
4. How might we connect and strengthen other parts of the system for the greatest potential? Who are the key actors we must engage?

After the workshop, these data from the small groups were synthesized by EI and BHC to identify patterns. As patterns emerged, they were crafted into 3–4 unique but mutually reinforcing “Leverage Opportunities,” visualized as an overlay to the context map in Kumu. All qualitative data collected during this workshop was synthesized into the leverage narratives, and the flags were stored as a map layer in Kumu.

### Step #3: Taking Action—Data Collection and Analysis Techniques

This final step again convened the same stakeholders into a workshop. Armed with the context map and leverage opportunities, stakeholders were reconvened to design a set of “Coherent Actions,” which are multi-sector actions that aim to address all of the leverage opportunities relating to one major urban health theme. Focusing on the intersecting patterns within the maps, EI and BHC asked the participants to use the results of the previous workshops to flesh out a set of, *“How might we…”* questions, which are meant to define those key patterns in the city system that can mitigate, disrupt, or transform the major inhibitors to reaching the city’s vision statement. The BHC facilitators strategically grouped participants by their expertise areas to discuss multi-sector actions addressing similar “How might we?” questions. EI coached the groups to use a facilitation tool called a prototype canvas that fleshed out details for each action.

**Fig. 5:**
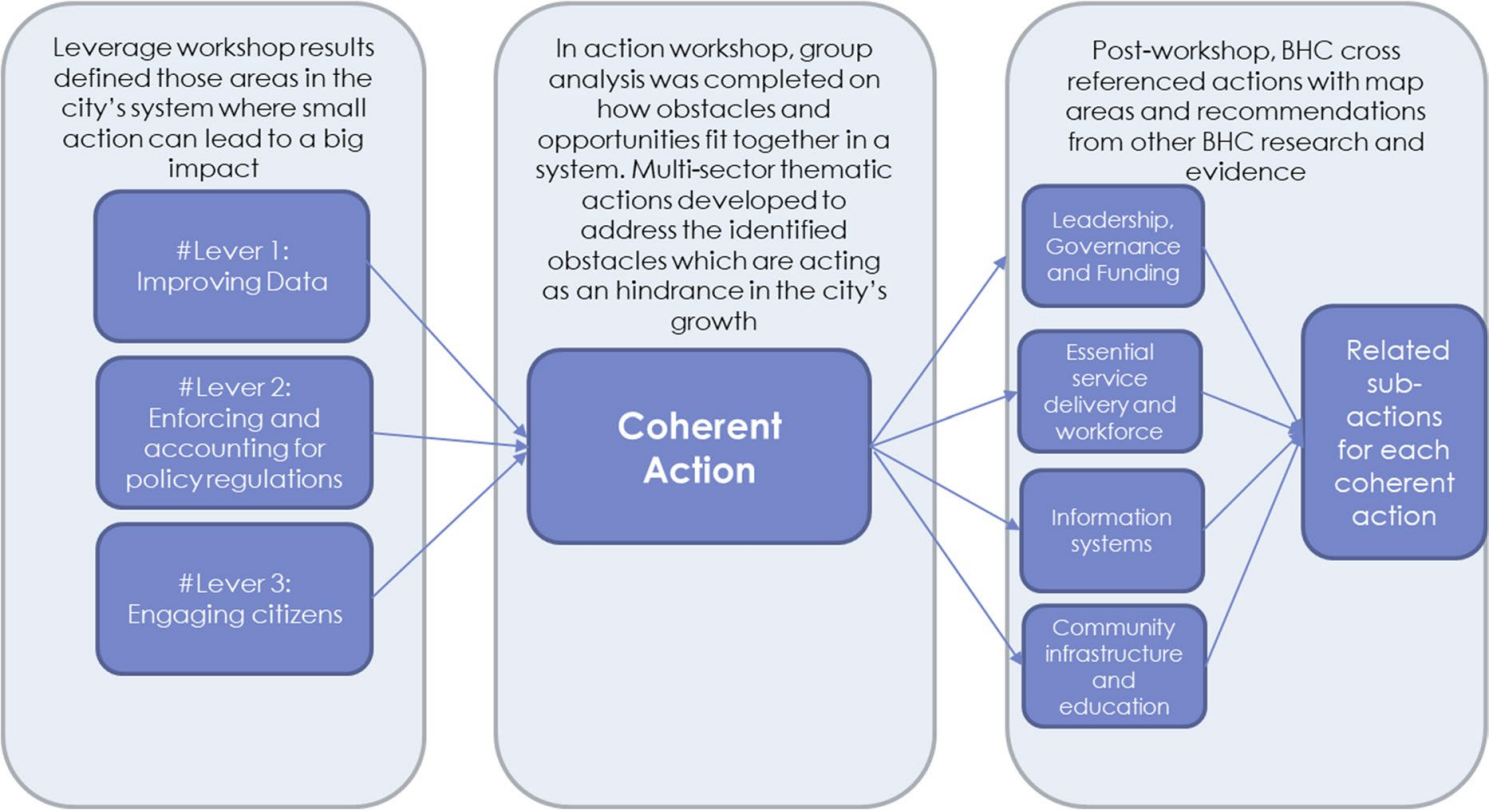
BHC’s triangulation method for completing coherent actions

BHC started the action planning step right as the COVID-19 pandemic began, which limited opportunities to safely share findings and receive feedback as a follow up on the coherent actions. As such, BHC triangulated the action planning workshop results with the project’s other work to complete a draft set of coherent actions. This process is described in Fig. 5.

This triangulation process resulted in additional coherent actions for each city, particularly around multi-sector management, funding, and coordination for activities. It also deepened the descriptions, recommendations, and implementation details for the existing coherent actions developed during the workshop. Data was not stored in Kumu for this phase, but instead was written into a Healthy City Action Plan report. These documents are further described in the city-specific articles in this issue.

## Discussion

BHC and EI’s experience provides a practical application of systems thinking methodologies in the context of municipal planning. In an era of renewed interest in multi-sector health, we believe this particular permutation of systems mapping provides a highly transparent, participatory, and understandable pathway to defining key priorities across sectors while providing a common goal for a diverse group that is sometimes at odds over resources. It is also flexible and adaptable to many situations. After BHC ends, the ease of public access to the Kumu software allows for a smoother transition of the maps to local ownership. The ability to regularly update the map with new data keeps the findings relevant and useful and reinforces data use in yearly workplanning. To our knowledge, no other systems mapping process has produced actual changes to funding and municipal planning functions. In BHC’s other articles in this issue, we describe how the methods described in this paper have resulted in some of those changes.

## Limitations and Considerations

As with any methodology, there are limitations that should be considered. This systems mapping model inherits both strengths and weaknesses from the fields of study from which it has evolved. Like any qualitative work, it is only as good or representative as the stakeholders and data included and depends on honest feedback. If the maps miss key perspectives or lack honesty, the visual appeal of the design can hide these gaps, as well as mass generalizations about the system. It is very important that those facilitating this process pay attention to both representation and the accuracy of the final loops and narratives. As with other community-based approaches, in certain contexts, stakeholders may place more value on short-term goals (or emergencies) versus long-term systems change. This does not mean the outcomes will be inaccurate, but if they are used for policy or planning purposes, the results may be myopic. Shortcomings specific to EI and BHC’s application are that the design was not originally built for a system as large as a city. EI and BHC had to evolve the method to ensure the maps represented themes that repeated across sectors, neighborhoods, and workforce cadres, so that the context map provided a more useful platform for leveraging citywide solutions. Finally, the action step was originally designed for organizational level action. BHC made adjustments to better fit a city planning context.

Another consideration is how to define this methodology for ethical review. When BHC and EI began the maps in our first two cities, we considered it a planning tool to be applied as part of the course of regular public health practice, and as such did not seek IRB clearance (though BHC did receive IRB exemptions for the baseline assessments that underpinned the context mapping). As work progressed, BHC saw the research value of the work and received IRB exemption in our third city, Da Nang. Identifying this as a research study in that context also helped us gain buy-in from certain stakeholders. In contrast, the stakeholders in BHC’s fourth city (Kathmandu) specifically did not want to participate in another study because they felt the research landscape was already saturated, but were willing to participate in systems mapping with the goal of how to operationalize what information they already had at hand. The flexibility of EI’s approach made it a useful tool in all four contexts, but BHC had to define it differently for each city to gain buy-in. Regardless of how it is categorized, this type of systems work always requires informed consent from participants, and protection of privacy and free speech for vulnerable populations that are included. In addition to these data collection safeguards, EI and BHC stored all data in a way that individual responses could not be identified.

If these considerations of quality, representativeness, and ethical responsibilities are kept in mind, this innovative mapping process is a powerful tool for defining municipal priorities within growing cities across the globe, where the situation is rapidly changing and evolving. It can be used to provide evidence-based information on where to invest to gain the biggest impact on specific goals.

## Conclusion

Donella Meadows, a leader in the field of systems thinking, teaches us that it is unwise to try to impose our will on systems; instead, we must learn to listen for the assets and energy that are already there and harness them to align with our shared values [56]. We hope sharing this methodology and the resulting impacts of this systems work (in our other articles) will encourage other cities to consider this or similar processes to help guide their healthy city planning.

## Data Availability

No data was collected specific to the creation of this methodology.
